# Machine learning techniques for identification of carcinogenic mutations, which cause breast adenocarcinoma

**DOI:** 10.1038/s41598-022-15533-8

**Published:** 2022-07-11

**Authors:** Asghar Ali Shah, Hafiz Abid Mahmood Malik, AbdulHafeez Mohammad, Yaser Daanial Khan, Abdullah Alourani

**Affiliations:** 1 Department of Computer Science, University of Management and Technology, Lahore, Pakistan; 2 Department of Computer Sciences, Bahria University Lahore, Lahore, Pakistan; 3 Faculty of Computer Studies, Arab Open University Bahrain, A’ali, Bahrain; 4 Department of Computer Science and Information, College of Science in Zulfi, Majmaah University, Al Majma’ah, Saudi Arabia

**Keywords:** Biotechnology, Cancer, Computational biology and bioinformatics, Health care, Computational science, Computer science

## Abstract

Breast adenocarcinoma is the most common of all cancers that occur in women. According to the United States of America survey, more than 282,000 breast cancer patients are registered each year; most of them are women. Detection of cancer at its early stage saves many lives. Each cell contains the genetic code in the form of gene sequences. Changes in the gene sequences may lead to cancer. Replication and/or recombination in the gene base sometimes lead to a permanent change in the nucleotide sequence of the genome, called a mutation. Cancer driver mutations can lead to cancer. The proposed study develops a framework for the early detection of breast adenocarcinoma using machine learning techniques. Every gene has a specific sequence of nucleotides. A total of 99 genes are identified in various studies whose mutations can lead to breast adenocarcinoma. This study uses the dataset taken from 4127 human samples, including men and women from more than 12 cohorts. A total of 6170 mutations in gene sequences are used in this study. Decision Tree, Random Forest, and Gaussian Naïve Bayes are applied to these gene sequences using three evaluation methods: independent set testing, self-consistency testing, and tenfold cross-validation testing. Evaluation metrics such as accuracy, specificity, sensitivity, and Mathew’s correlation coefficient are calculated. The decision tree algorithm obtains the best accuracy of 99% for each evaluation method.

## Introduction

Machine Learning plays a phenomenal role in solving many crucial issues in various fields of life. Adenocarcinoma is a type of cancer that begins in secretary cells. Breast Adenocarcinoma is the abnormal and uncontrolled growth of cells in the breast gland. It is the second most severe cancer among all the cancers present in the human body.

It mostly occurs in women. An estimated 0.3 million women are diagnosed with breast cancer each year in the United States of America. In 2021 estimated 44,130 deaths (43,600 women and 530 men) occurred due to breast cancer in the United States^1^. There are several reasons for breast cancer in women. Some are getting older with age, having a family breast cancer history, having a child after 35, starting menopause after 55, having high bone density, etc.

**Figure 1:**
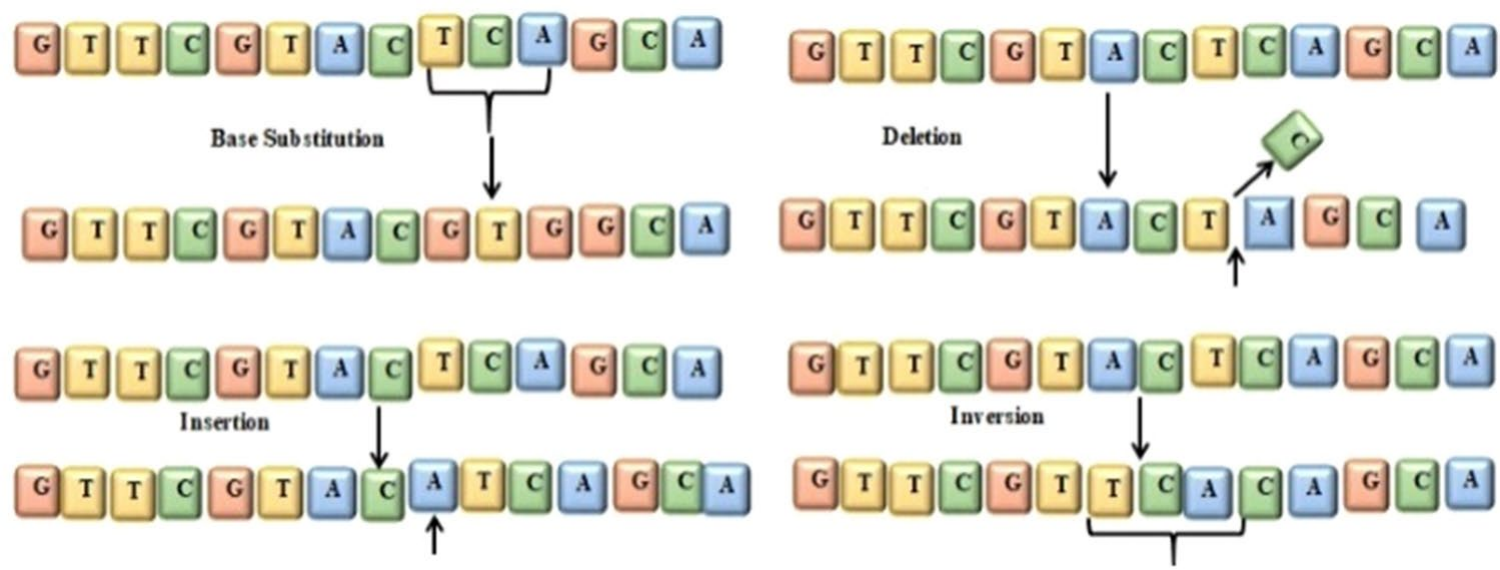
A point mutation in a gene.

A Biopsy is a primary technique used for the detection of breast adenocarcinoma. It is the technique in which a small tissue is examined under a microscope^2^. The Artificial intelligence (AI) approach has potential effects in the field of medical science. Several AI techniques are used in the medical science field to detect various diseases inside the human body. In this study machine learning approach is used to detect breast cancer at its early stage. There are sequences of nucleotides in the human gene. Any change in the sequence is called a mutation, which mostly leads to cancer^3^. Figure 1 illustrates the process of mutation.

DNA is a sequence of 4 basic nucleotides Adenine (A), Guanine (G), Thymine (T), and Cytosine (C)^4^. Any change in the base sequence in genes leads to mutation. This change may be caused by insertion, deletion, or replication of the gene base and may cause damage to DNA. Different factors affect DNA. These factors include metabolic activities or environmental factors such as radiation, resulting in tens of thousands of individual DNA damage per cell per day^5^. The DNA molecule’s damage alters or eliminates the cell’s ability to transcribe the gene. DNA repair is when a cell identifies and corrects damage to the DNA^6^. This process is constantly active as it responds to damage in the DNA structure. When normal repair processes fail, the cellular apoptosis is disrupted, and DNA damage may not be repairable. This irreparable damage leads to malignant tumors or cancer as per the two-hit hypothesis^7,8^.

This study uses a machine learning framework for the identification of breast adenocarcinoma. Three machine learning algorithms: Decision tree, Gaussian Naïve Bayes, and Random Forest, are applied to three evaluation methods: self-consistency test, independent set test, and tenfold cross-validation test. After using the machine learning algorithms on evaluation methods, accuracy, specificity, sensitivity, and Mathew’s correlation coefficient is calculated, explained in the results and discussion section.

## Literature review

**Figure 2:**
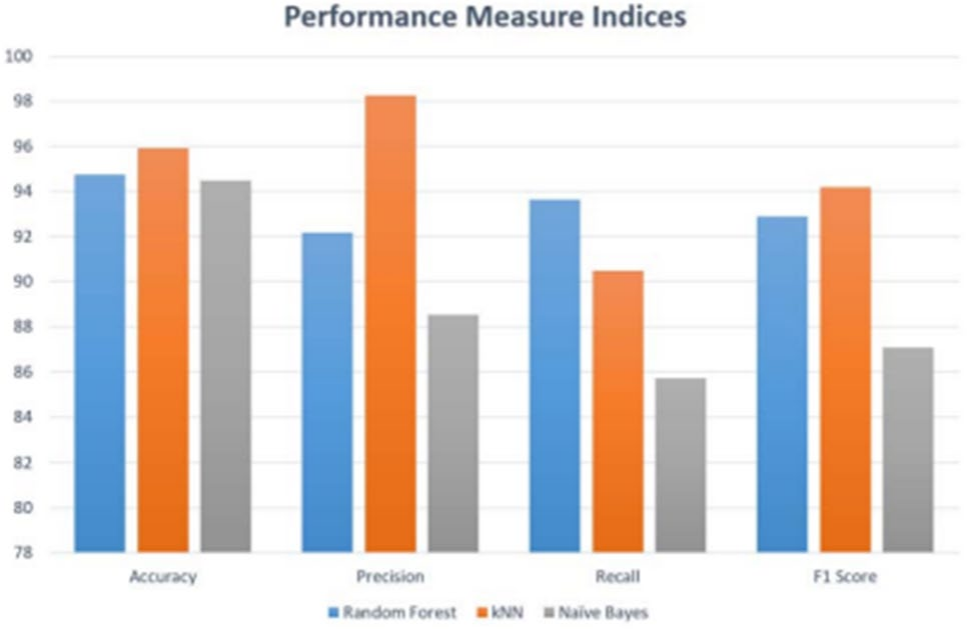
Performance measure of machine learning algorithms.

Breast cancer is the second major cause of death in women. In 2021 estimated 44,130 deaths occurred due to breast cancer in the United States^9^. Breast cancer was discovered in the early 400 s B.C.E^10,11^. Breast cancer develops from breast tissue^12^. Breast cancer is a genetic disorder, and the development of breast cancer has a genetic component^13–16^. Breast adenocarcinoma develops in cells from the lining of milk ducts and lobules. Lobules supply these ducts with milk^12^. Breast cancer is more common in developed countries^13^. Oncogenomics mutations lead to the uncontrollable growth of cancer cells. Although every mutation in the sequence doesn’t cause cancer, every cell growth is not cancerous. The interruption in the balance of creating cells and destruction of cells causes the beginning of cancer. This happens because of the change in the functional characteristics of genes. Cancer driver genes drive the development of cancer. Therefore, the mutation caused in driver genes commonly leads to cancerous mutation while passenger mutations do not cause cancer^2,17^. Driver and passenger mutations have been identified by various researchers based on clinical data. A well-known database of cancer driver genes called IntoGen reports that there are 99 driver genes that can cause breast adenocarcinoma which is a malignant tumor^18^. Bioinformatics plays a vital role in the field of medical sciences. There are many machine learning and computational technologies used in the area of medicine for the detection and prevention of various diseases. A study conducted by Botstein et al. used a semi-supervised approach in 2000 to identify the subtypes of breast cancer but initialing curating a database of the genes involved in breast cancer^19^. There are many machine learning algorithms developed for breast adenocarcinoma detection and identification. In research, the leveraging Machine Learning algorithm is applied to the dataset of 683 patients taken from the M. G Cancer Hospital & Research Institute, Visakhapatnam, India. The dataset is preprocessed using Gaussian filters, and then the features are extracted^20^. The accuracy in detecting breast cancer by the Deep Neural Network with Support Value (DNNS) model was 97.21%. Researchers have employed different machine learning algorithms, including artificial neural networks (ANNs), support vector machines (SVMs), decision trees (DTs), and k-nearest neighbors (k-NNs) applied to the Wisconsin breast cancer database (WBCD) dataset for the detection of breast cancer^21^. Data mining also plays an important role in the detection of breast cancer. Data mining techniques are applied to Decision tree, Naïve Bayes, and Sequential Minimal Optimization algorithms^9^. Subsequently, random forest (RF), k-NNs, and Naïve Bayes (NB) algorithms were applied to the WBDC dataset. The accuracy of k-NNs, Random forest, and NB was 95.90%, 94.74% 94.47%, respectively. Figure 2 explains the performance measure for this work^22^.

Another technique used is the Fast Correlation-Based Filter (FCBF) method to predict and classify breast cancer. Five machine learning algorithms are applied in RF, SVM, k-NNs, NB, and multilayer perceptron (MLP). The highest accuracy for this system is given by SVM. The Accuracy of SVM is 97.9%^23^. The extended form of the Naïve Bayes algorithm, the Weighted Naïve Bayes algorithm, is applied to the UCI machine learning breast cancer dataset for breast cancer prediction. The accuracy of this model was 92%^24^. Another similar study was conducted in 2021^25^, which implemented RF, SVM, and ANN and achieved accuracy, sensitivity, and specificity in an independent set test such as 91.06%, 81.27, and 87.26, respectively.

## Methodology

**Figure 3:**
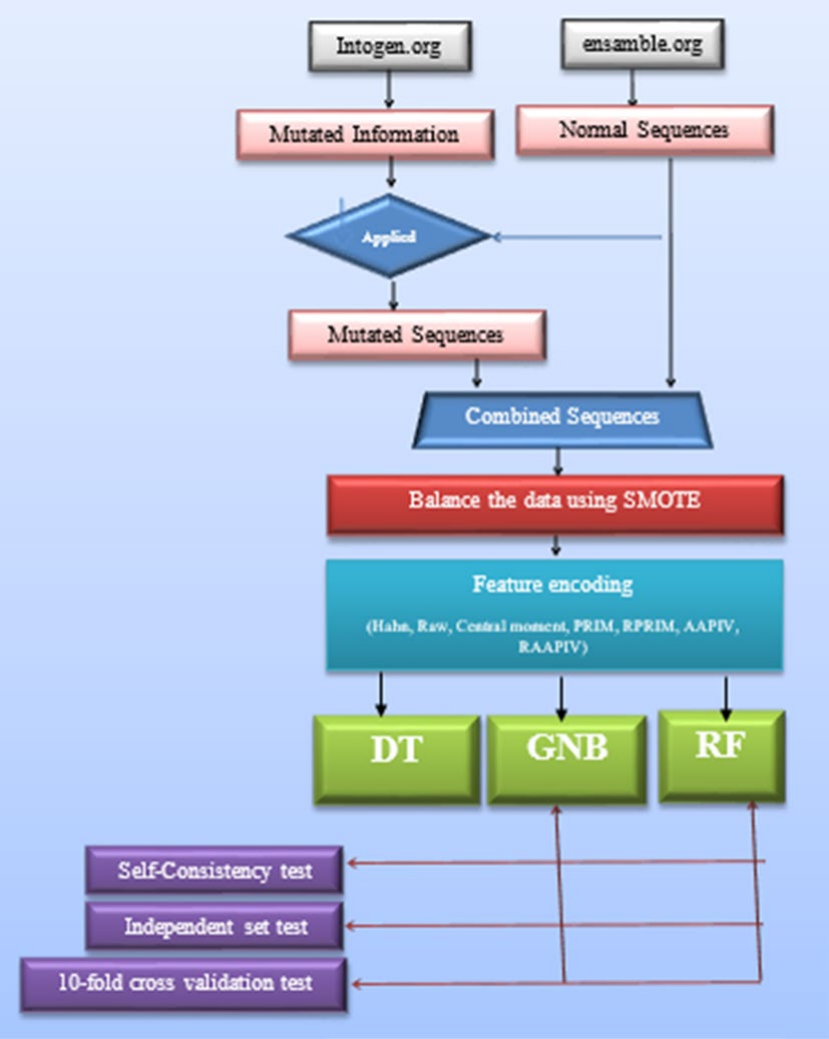
Systematic diagram of the proposed model.

This study uses machine learning techniques for the detection of Breast Adenocarcinoma. Different machine learning algorithms are involved in the study to identify cancer. The systematic diagram of the proposed system is shown the Fig. 3.

Figure 3 explains the working of the complete process step by step. Decision tree, Random Forest, and Gaussian Naïve Bayes are used in each evaluation method to identify mutation for detecting breast adenocarcinoma. Researchers can use the proposed framework to develop an early warning diagnostic system based on genomic data. It will enable oncologists to detect and treat breast adenocarcinoma more personalized. The following sections explain the algorithms in detail with their testing methods and ROC curves.

### Benchmark dataset collection

The dataset is the most critical factor for any bioinformatics related study. Typically, the dataset is used for training, testing, and validation. This study aims to use a high-quality benchmark dataset that is highly accurate and relevant to the study to obtain the best results. A meaningful dataset of the Breast Adenocarcinoma driver gene sequences is selected. The normal gene sequences are taken from https://asia.ensembl.org/^26^. Mutation pieces of information are taken from the most recent version available on http://intogen.org/^18^. IntOgen database does not contain mutated sequences. It has only mutation information. Therefore an application is developed in python to incorporate this information in normal gene sequences, taken from https://asia.ensembl.org/, to construct mutated sequences. The passenger mutations are not carcinogenic; therefore, these are considered normal sequences. Driver mutations are carcinogenic mutations. For the proposed study, 4127 human samples are used with a total of 6170 mutations in a total of 99 genes involved in breast adenocarcinoma. Genes involved in Breast Adenocarcinoma are shown in Table 1.

**Table 1:** Genes involved in breast adenocarcinoma and mutation.

Gene symbol	Mutations	Samples	Gene symbol	Mutations	Samples
TP53	846	820	HOXC13	9	8
PIK3CA	866	802	CACNA1D	53	8
KMT2C	205	184	ELN	26	8
GATA3	63	179	ZXDB	18	8
CDH1	176	176	NTRK1	26	8
MAP3K1	126	149	SALL4	17	7
ESR1	129	108	NOTCH2	75	7
PTEN	105	105	PDGFRB	17	7
AKT1	88	86	SMAD2	17	7
NCOR1	89	76	EPAS1	25	7
ARID1A	76	76	RHPN2	18	5
MAP2K4	72	75	SMAD4	17	5
FOXA1	72	70	MAX	9	5
TBX3	54	65	HRAS	10	5
NF1	85	65	ZFHX3	72	5
ERBB2	83	60	ERBB4	43	4
RB1	60	55	CUX1	29	4
CBFB	64	53	MDM4	14	4
SF3B1	56	50	KLF4	8	4
KMT2D	99	43	GATA1	15	4
FAT3	112	40	HOXD13	10	4
ERBB3	55	39	FAT1	61	4
PREX2	73	39	USP6	19	4
CTCF	47	37	DDX3X	23	4
LRP1B	114	36	EGFR	45	3
RUNX1	37	35	NONO	9	3
PIK3R1	24	32	MEN1	28	3
ATM	64	29	MTOR	57	3
SPEN	74	29	GNAS	29	3
FGFR2	37	27	ASXL1	36	3
BRCA1	49	23	KDM6A	30	3
CASP8	28	20	FAT4	78	3
CREBBP	48	17	ARHGEF12	26	3
FBXW7	29	17	MYO5A	19	3
BRCA2	52	16	POLD1	18	3
PTPRD	64	16	KAT6B	37	2
MYH11	59	16	PLAG1	15	2
RGS7	32	15	HSP90AA1	17	2
CDKN2A	17	15	ZBTB16	11	2
KRAS	15	13	ARID1B	46	2
NCOA1	21	13	JAK2	19	2
MYH9	59	12	NIN	44	2
PTPN13	42	12	ALK	33	2
ABL2	31	12	NUMA1	33	2
CDKN1B	19	11	SMARCD1	12	2
EPHA3	31	11	GRIN2A	48	2
NCOR2	44	11	BAP1	25	2
AFF3	37	10	CLTC	31	2
ETV5	16	9	HOXC13	9	8
BRAF	22	9	CACNA1D	53	8
HIST1H3B	27	14			

**Figure 4:**
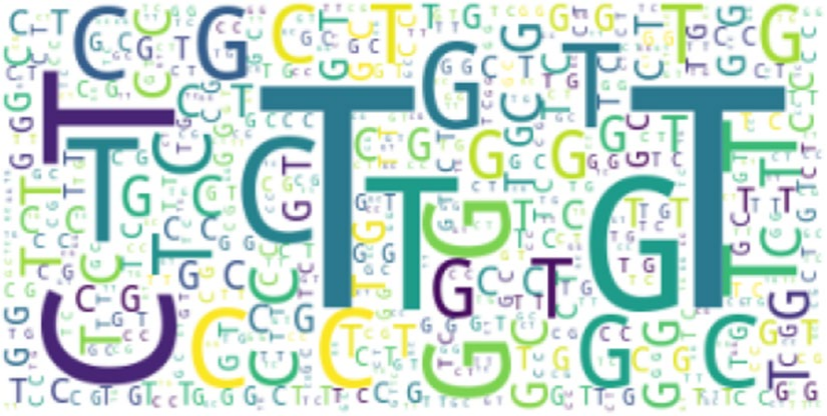
Word cloud of Breast adenocarcinoma dataset.

Word cloud is a visualization technique in python to represent text data. The size of each word indicates its frequency and importance^27^. The word cloud in Fig. 4 shows the frequency and importance of each nucleotide in all gene sequences related to breast adenocarcinoma.

### Synthetic minority over-sampling technique (SMOTE)

The SMOTE technique balances the dataset. An unbalanced dataset is a dataset in which classification is not equally represented. There are two standard techniques used to balance datasets oversampling and undersampling. In the under-sampling technique, the number of classes reduces to balance the dataset. The overall data records are reduced. While in the over-sampling technique, the number of minority classes is increased. Smote is an oversampling technique for balancing the dataset. SMOTE randomly selects the instances from the minority class. It uses the interpolation method to generate instances between the selected point and its nearby instances.

The steps involved in SMOTE algorithm are as follow^28^:

1. Insert dataset and mark minority and majority classes from it.
2. Calculate the number of instances generated from the percentage of oversampling.
3. From minority classes, identify random instance $K$ and find its neighbors $N$.
4. From any neighbors, find the difference between $N$ and $K$.
5. Multiply the difference with any number between 0 to 1 and add this difference to $K$.
6. Repeat the process until the required number of instances are generated.

**Figure 5:**
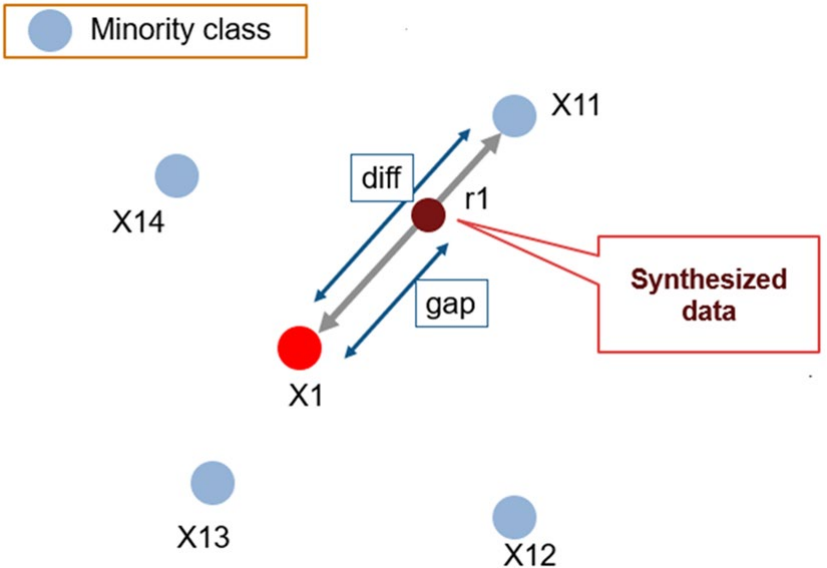
Creation of synthetic data points in SMOTE.

Figure 5 explains creating synthetic data points in SMOTE^29^.

The dataset for the proposed study is represented by a $B$ defined by Eq. (1).

1 $${\text{B}=\text{ B}}^{+ } U {\text{B}}^{- },$$

Here $B+$ are the mutated gene sequences that cause cancer while $B-$ are the normal gene sequences, and U is the union for both sequences.

### Feature extraction

Here H defines the gene sequence^25^.

The following Eqs. (2) and (3) calculate Hahne’s was polynomial.

2 $${h}_{n}^{r,s}\left(P, Q\right)= {\left(Q+V-1\right)}_{n}({Q-1)}_{n}\times \sum\limits _{z=0}^{n}{\left(-1\right)}^{z}\frac{{\left(-n\right)}_{z}{\left(-p\right)}_{z}{\left(2Q+r+s-n-1\right)}_{z}}{{\left(Q+s-1\right)}_{z}{\left(Q-1\right)}_{z}} \frac{1}{z!}.$$

Here $P$ is an integer value from any *Q*, $Q-1$ positive integers^31^. Hahn moment for two-dimension data is found by Eq. (3).

3 $$The {H}_{xy}= \sum \nolimits_{j=0}^{G-1}\sum \nolimits_{i=0}^{G-1}{\delta }_{xy}{h}_{x}^{a,b}{\left(j, Q\right)h}_{y}^{a,b}\left(j,Q\right),\quad m, n=0, 1, 2, . . ., Q-1.$$

The raw moment is used for data imputation. Imputation replaces the missing data values in the dataset with most substitute values to preserve the information^32^. The raw moment for the 2D data with order $a+b$ is expressed by Eq. (4)^33^.

4 $${U}_{ab}= \sum \nolimits_{e=1}^{n}\sum \nolimits_{f=1}^{n}{e}^{a}{f}^{b}{\delta }_{ef}.$$

Centroids $(r, s)$ are required to compute the central moments visualized as the center of data. By exploiting the centroids, central moments can be computed as.

5 $${V}_{rs}= \sum \nolimits_{e=1}^{n} \sum \nolimits_{f=1}^{n}{\left(e-\overline{x }\right)}^{r} {\left(f-\overline{y }\right)}^{s} \delta ef.$$

Position Relative incidence matrix (PRIM) is used to determine each gene’s positioning in the gene sequence of breast adenocarcinoma. PRIM formed matrix with the dimension of 20 by 20 is shown in Eq. (4)^34^.

6 $${R}_{PRIM}= \left[\begin{array}{ccc}\begin{array}{cc}{R}_{1\to 1}& {R}_{1\to 2\cdots }\\ {R}_{2\to 1}& {R}_{2\to 2\cdots }\end{array}& \begin{array}{c}{R}_{1\to q\cdots }\\ {R}_{2\to q\cdots }\end{array}& \begin{array}{c}{R}_{1\to M}\\ {R}_{2\to M}\end{array}\\ \begin{array}{cc}\vdots & \vdots \\ {R}_{p\to 1}& {R}_{p\to 2\cdots }\end{array}& \begin{array}{c}\vdots \\ {R}_{p\to q\cdots }\end{array}& \begin{array}{c}\vdots \\ {R}_{p\to M}\end{array}\\ \begin{array}{cc}\vdots & \vdots \\ {R}_{M\to 1}& {R}_{M\to 2\cdots }\end{array}& \begin{array}{c}\vdots \\ {R}_{M\to q\cdots }\end{array}& \begin{array}{c}\vdots \\ {R}_{M\to M}\end{array}\end{array}\right].$$

Feature scaling allows each data sample to participate in detecting breast cancer^30^. In machine learning, the algorithm is considered more efficient in which the most relevant data has been extracted. PRIM did not extract all the information from the data. Reverse Position Relative incidence matrix (RPRIM) also works the same as PRIM works but in the reverse sequence.

The frequency matrix provides information about the occurrence of genes in the gene sequence. The accumulative absolute position incidence vector (AAPIV) includes information about the composition of the gene sequence. The relative positioning of the cancer gene is found by using AAPIV. Equation (7) illustrates the relative positioning of the gene sequences^35^.

7 $${\text{AAPIV}} = \left\{ {\upvarepsilon_{1} ,\;\upvarepsilon_{2} ,\;\upvarepsilon_{3} , \ldots \upvarepsilon_{N} } \right\}.$$

The reverse Accumulative absolute position incidence vector (RAAPIV) works the same as AAPIV works but in the reverse order. The Eq. (8) for RAAPIV is as follows

8 $${\text{RAAPIV}} = \left\{ {\upvarepsilon_{1} ,\;\upvarepsilon_{2} ,\;\upvarepsilon_{3} , \ldots \upvarepsilon_{N} } \right\}.$$

### Prediction algorithms

This study uses a Decision Tree, Gaussian Naïve Bayes, and Random Forest Classifier for the prediction of Breast Adenocarcinoma.

The decision tree is a supervised machine learning technique. It is mostly used for classification and regression problems. In a decision tree root, nodes can be used as input. These nodes are filtered through decision nodes and leaf nodes used for getting desired output^35–37^. Entropy controls how data will be split in the decision tree, and information gain tells how much information a feature gives about the respective class. Equations (9) and (10) explain the formula for calculating Entropy and information gain in the decision tree^38^.9

10 $${\text{IG}} = {\text{ Entropy }}\left( {{\text{Parent}}} \right) \, - {\text{ Average Entropy }}\left( {{\text{Child}}} \right).$$

**Figure 6:**
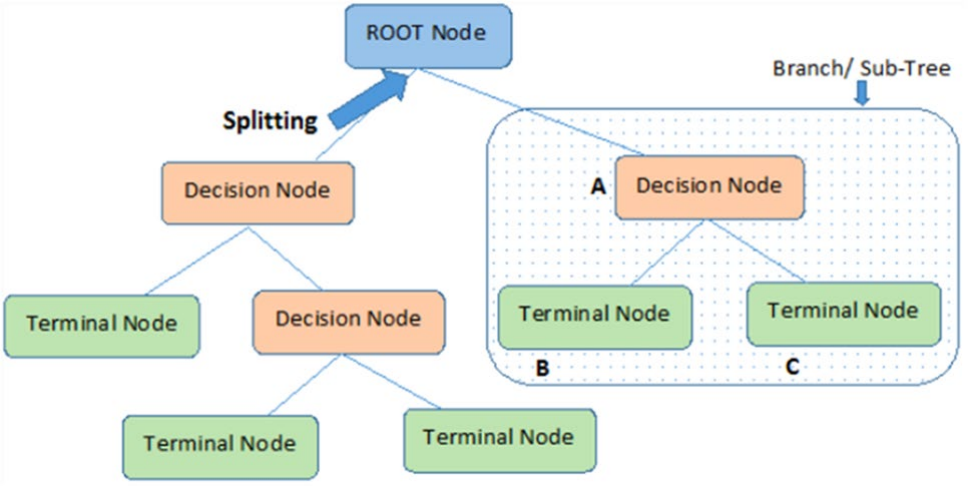
Nodes of decision tree.

In the decision tree, the data flow in nodes. Figure 6 explains the working of the decision tree algorithm^39^.

The Naive Bayes algorithm is mostly used in data mining algorithms based on the Bayes theorem and uses simple probabilities. The Eq. (11) of Bayes theorem is as follows^40^.

11 $$P\left(B|Y\right)= \frac{P\left(B\right)P\left(B|Y\right)}{P|Y}.$$

**Figure 7:**
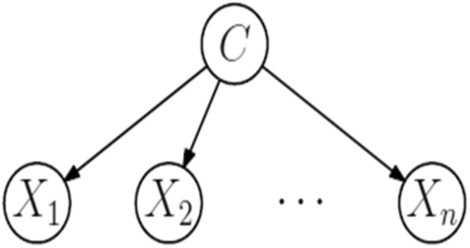
Naïve Bayes classifier.

Here P refers to probability, and Y is the attribute of a class. Figure 7 explains the Naïve Bayes classification^41^.

The algorithm for NB is
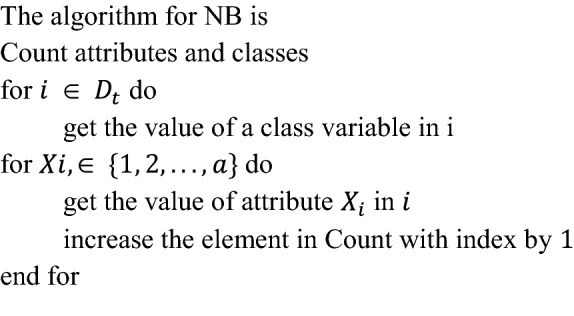


Here ${D}_{t}$ is the set of training examples, $i$ is the instance, and ${X}_{i}$ is the random variable^42^. It is an easy algorithm used in many fields of medical science^43^.

**Figure 8:**
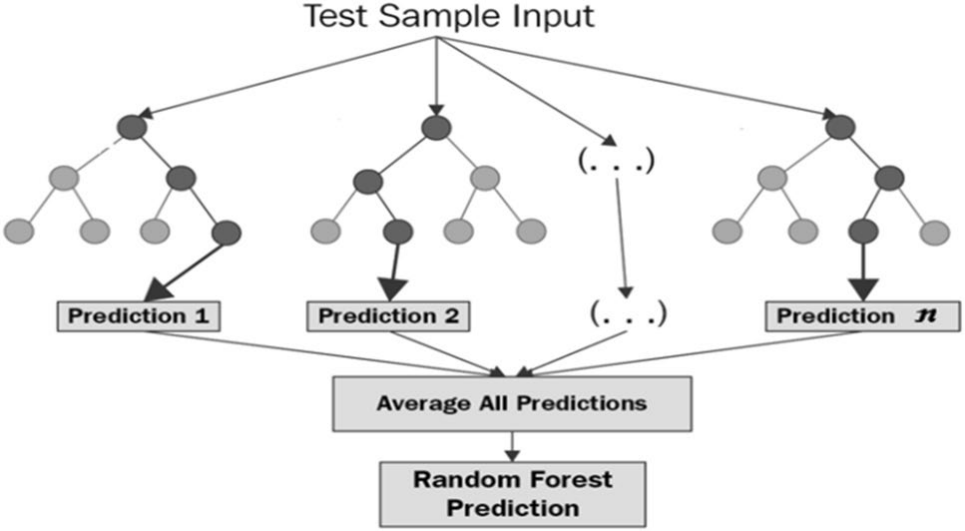
Working on Random Forest algorithm.

Random Forest (RF) is the third algorithm applied to all the evaluation methods. It is the collection of the tree predictions which use different data for different techniques, and each technique leads to a different result. It is the ensemble learning method for regression and classification by constructing a multitude of decision trees^44^. The result is merged to represent the average result. Figure 8 illustrates the working of Random forest algorithms^45^.

MSE measures the average of the square of errors. It is the difference between the actual values and calculated values. The mean square error in RF is measured by Eq. (12).

12 $$MSE = \frac{1}{{{N}}}\sum \nolimits_{I=1}^{N}{({f}_{1}-{y}_{1})}^{2}.$$

In the equation ${({f}_{1}-{y}_{1})}^{2}$, is the square of errors.

Where ${y}_{1}$ is the predicted values and ${f}_{1}$ is the actual values.

13 $$Entropy = \sum \nolimits_{I=1}^{C}-{p}_{1}\times {\text{log}}_{2}{p}_{1}.$$

Entropy is used to measure uncertainty and disorder. In Eq. (13), p1 is the prior probability of each class, c, and the number of unique classes^46^.

## Results

Four types of evaluation methods are applied for the proposed research. The DT, GNB, and RF results are discussed in this section. For each technique, accuracy, specificity, sensitivity, Mathew's correlation coefficient, and accuracy is measured by the following equations^47–49^.

14 $$\text{Sensitivity }= \frac{\text{TPV}}{\text{TPV}+\text{FNV}},$$

15 $$\text{Specificity}= \frac{\text{TNV}}{\text{TNV}+\text{FPV}},$$

16 $$\text{Accuracy}= \frac{\text{TPV}+\text{TNV}}{\text{TPV}+\text{FPV}+\text{FNV}+\text{TNV}},$$

17 $$\text{Mathew's correlation coefficient }= \frac{\left(\text{TPV}\times \text{TNV}\right)-(\text{FPV}\times \text{FNV})}{\sqrt{(TPV+FPV)(TPV+FNV)(TNV+FPV)(TNV+FNV})}.$$

In the equations: $TPV$ = All the true positive values from the dataset, $TNV$ = All the True Negative values, $FNV$ = False Negative values, $FPV$ = False positive values.

For the proposed study, sensitivity refers to the ability of tests that truly identify Breast Adenocarcinoma cancer. Specificity refers to the tests’ ability to truly identify those who did not have Breast Adenocarcinoma in the dataset^40^. $TPV + FNV$ represents the total number of subjects with the given conditions in the equations. In comparison, $TNV+FPV$ is the total number of subjects without disorders. $TPV+FPV$ is the total number of subjects with positive tests, and $TNV+FNV$ is the total number of negative results^50^.

### Self-consistency test

It is the iterative process that stops when the test results are satisfied. The same data is used in this technique for training and testing purposes. Table 2 shows the Decision tree results, Gaussian Naïve Bayes, Random Forest of Breast Adenocarcinoma cancer while the self-consistency test is applied to it.

**Table 2:** Results of self consistency test.

	DT	GNB	RF
Accuracy	99%	81%	97%
Sensitivity	99%	79%	99%
Specificity	100%	84%	92%
Mcc	0.50	0.31	0.68

**Figure 9:**
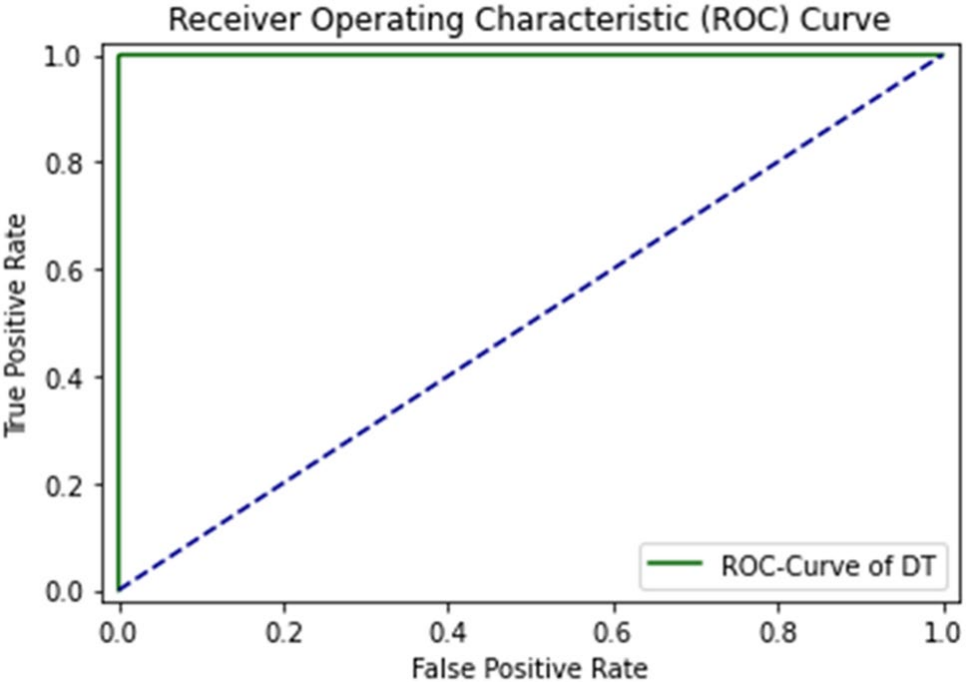
ROC curve of DT using self consistency test.

ROC Curve of DT using self-consistency test is shown in Fig. 9.

**Figure 10:**
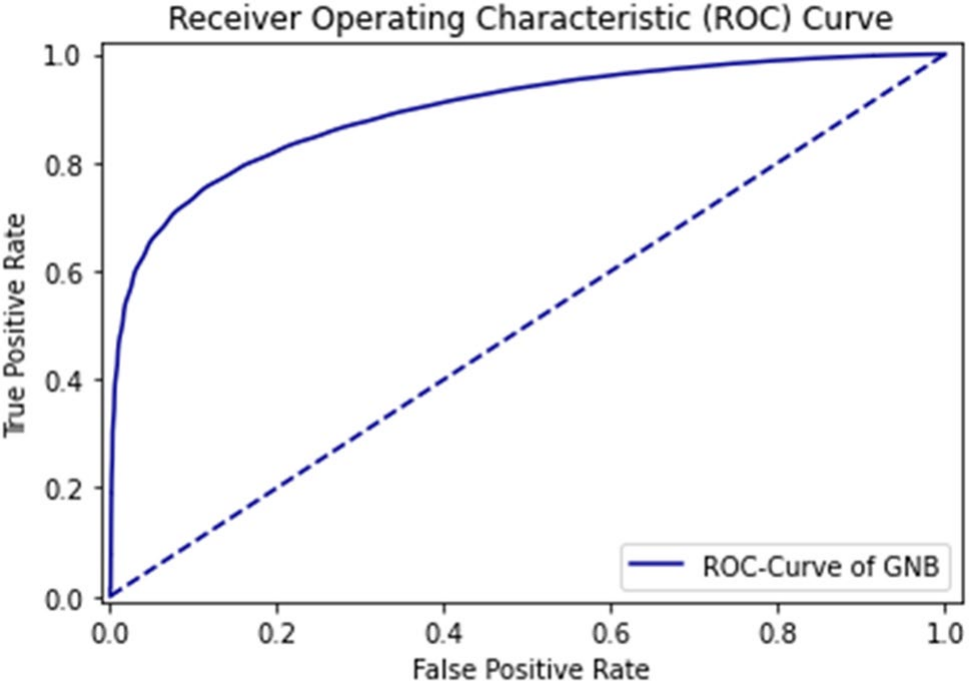
ROC curve of GNB using self consistency test.

The ROC curve defines the result as between 0.99 and 1.0, which should be considered excellent. ROC Curve of GNB using self-consistency test is shown in Fig. 10.

**Figure 11:**
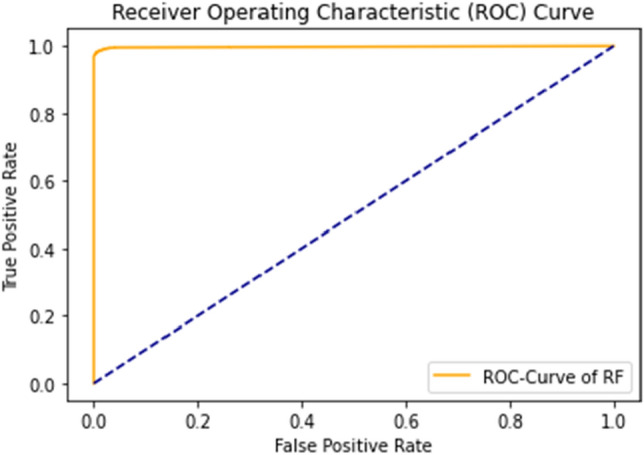
ROC curve of RF using self consistency test.

The rapid increase in the curve shows the accuracy value increases rapidly. ROC Curve of RF using the self-consistency test is shown in Fig. 11.

**Figure 12:**
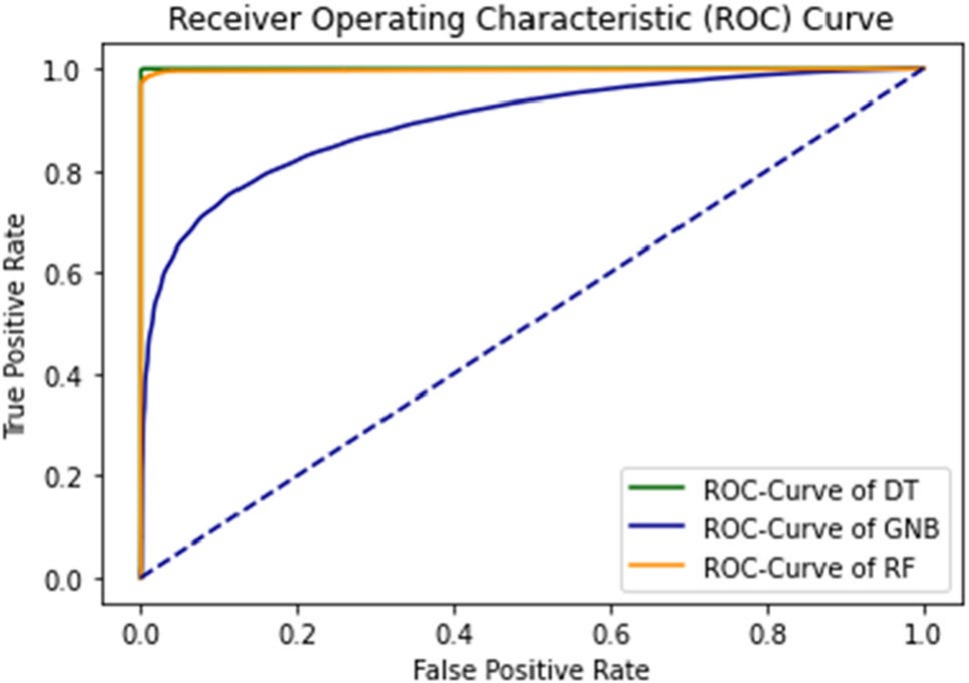
Combined ROC curve of self consistency test.

The combined ROC curve of the self-consistency test is shown in Fig. 12.

The ROC curve represents that all the results are on the upper side of the diagonal (50%), which shows the results under consideration are the best results. A Decision tree of 99% achieves the best outcome for the self-consistency test.

### Independent set testing

The first evaluation method for the proposed work is independent set testing. The values extracted from the confusion matrix are used to determine the model's accuracy. This test is the basic performance measuring method for the proposed model. 80% of values are used to train the algorithm from the dataset, and 20% are used for testing purposes. Table 3 illustrates the independent test results on DT, GNB, and RF.

**Table 3:** Results of independent set testing.

	DT	GNB	RF
Accuracy	99%	81%	95%
Sensitivity	99%	79%	99%
Specificity	99%	84%	92%
Mcc	0.70	0.70	0.67

**Figure 13:**
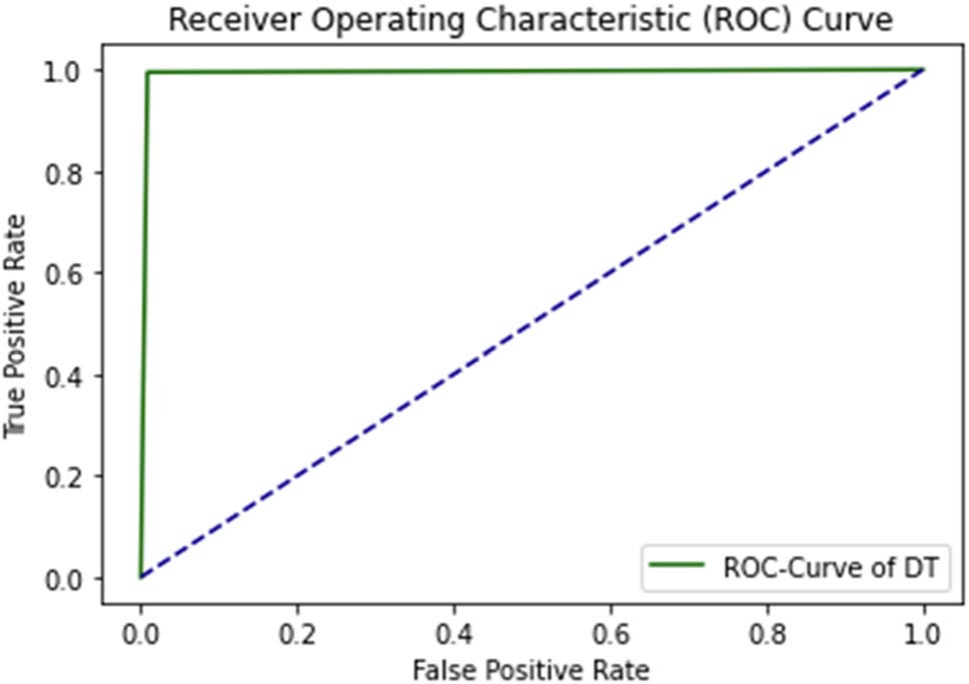
ROC curve of DT using independent set testing.

**Figure 14:**
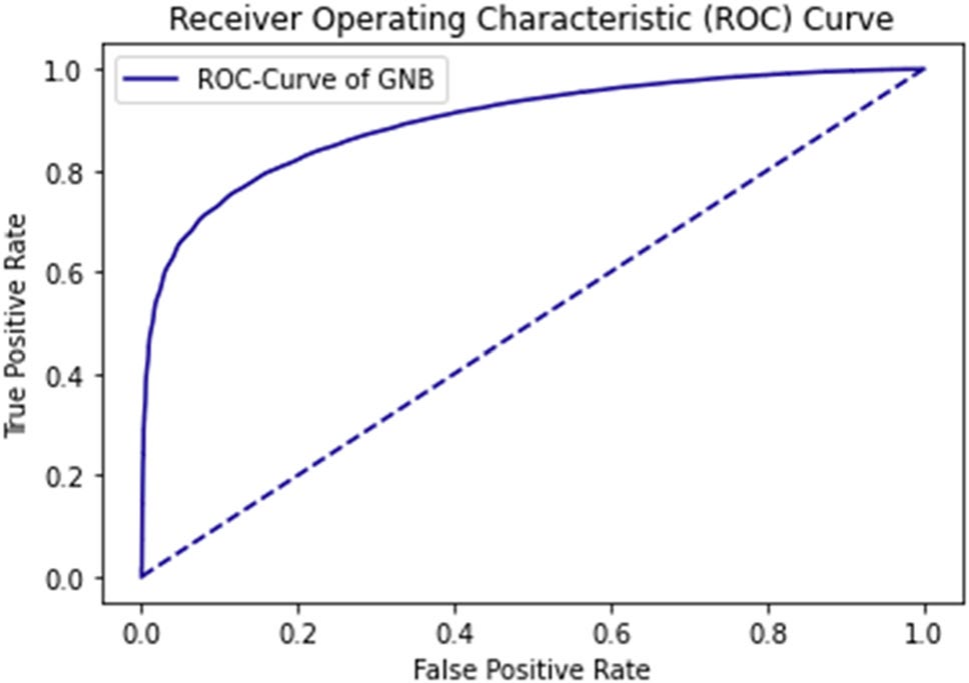
ROC curve of GNB using independent set testing.

**Figure 15:**
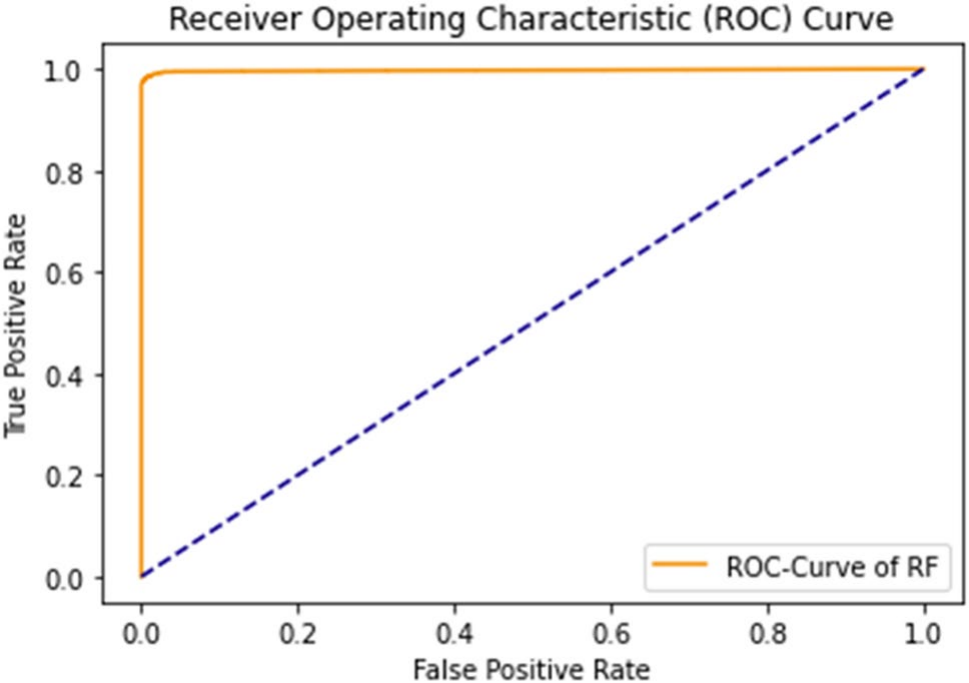
ROC curve of RF using independent set testing.

The Receiver Operating Characteristic (ROC) curve of DT, GNB, and FR Implemented after applying independent set testing is shown in Figs. 13, 14 and 15, respectively.

The ROC curve shows the specificity of 0.99 on the graph. It is the false-positive values get from the dataset. When sensitivity against the specificity together is a plot on the graph, a point in the ROC space is got. The position from the point in the ROC space shows the transaction between sensitivity and specificity. For most conditions, this point is between the points 0 and 1 on the graph. If this point falls on the area above the diagonal (more than 50%), it represents a good result; otherwise, it will be considered a bad result^51^. From the ROC curve of DT, it is determined that the point is falling above the diagonal at the point at 0.99, so it will be considered an excellent result.

The ROC curve of GNB shows the relation between sensitivity and specificity. The curve falls inside the ideal coordinate (0, 1). The result represents the value is between 0.8 and 0.85.

The ROC curve of RF shows a rapid increase in the results. The accuracy for this curve is 0.95.

**Figure 16:**
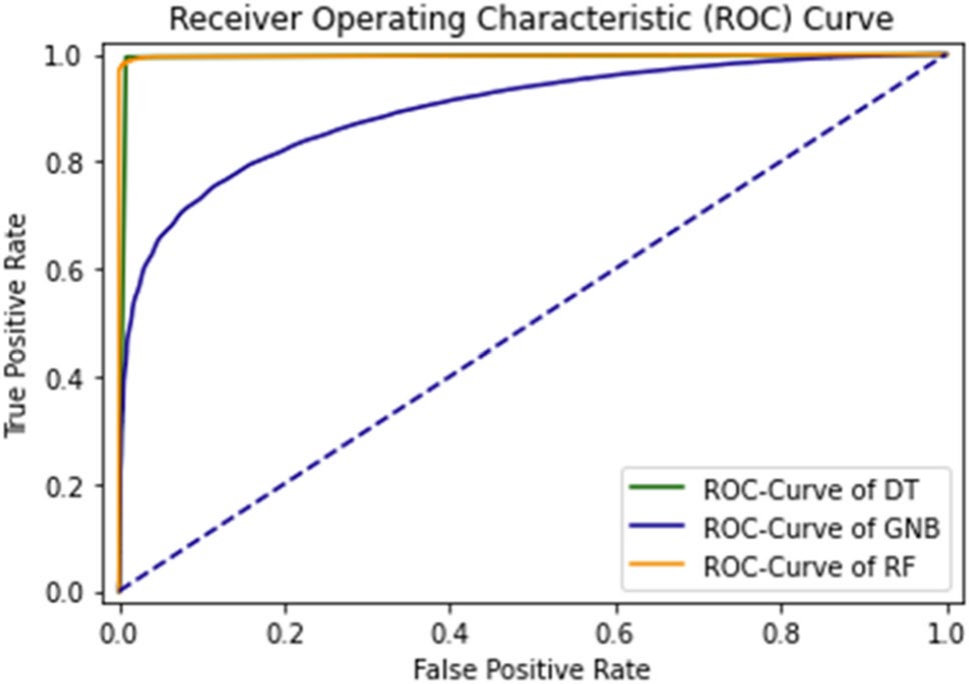
Combine the ROC curve of DT, GNB, and RF using independent set testing.

The combined ROC Curve of independent set testing is shown in Fig. 16.

The green graph line represents the curve of DT; the Yellow line shows the RF and the Blue line results for GNB. The Decision tree algorithm obtains the best accuracy.

### Tenfold cross-validation test

**Figure 17:**
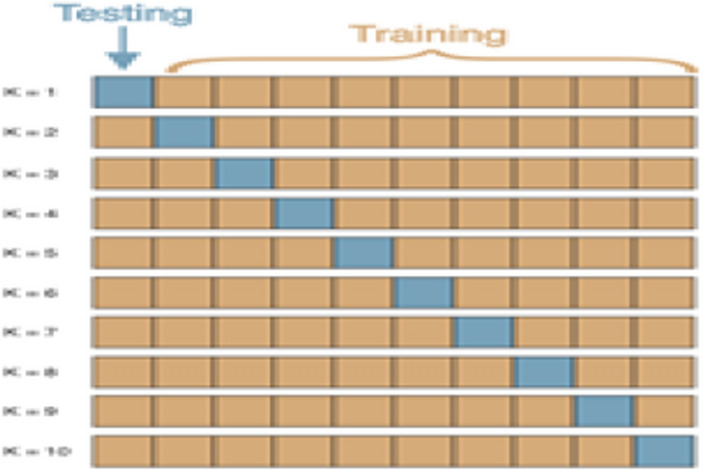
Working process of tenfold cross-validation.

The data is equally subsampled into ten groups in the tenfold cross-validation technique. Divide the training set into ten partitions and then treat each in the validation set, train the model, and average generalization performance across the tenfold to make choices about hyperparameters and architecture. Figure 17 shows the working process of the tenfold cross-validation technique.

Table 4 represents the result of the tenfold cross-validation technique.

**Table 4:** Results of tenfold cross-validation.

	DT	GNB	RF
Accuracy (acc)	99%	85%	92%
Sensitivity (sn)	98%	76%	85%
Specificity (sp)	99%	81%	99%
Mathew’s correlation coefficient (MCC)	0.98	0.62	0.85

**Figure 18:**
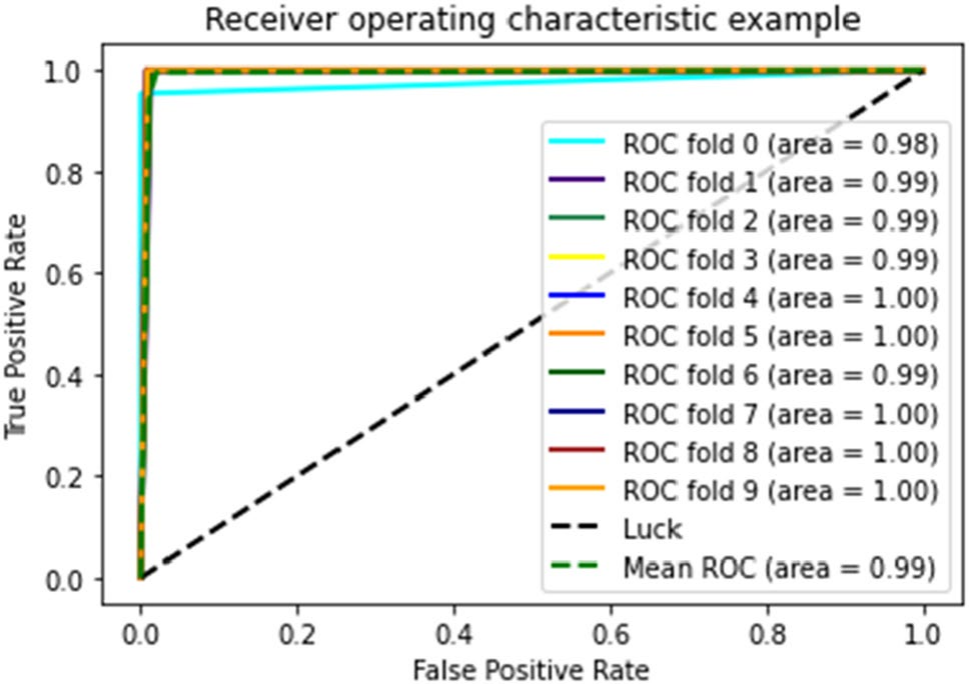
ROC curve of tenfold cross-validation applied on DT.

**Figure 19:**
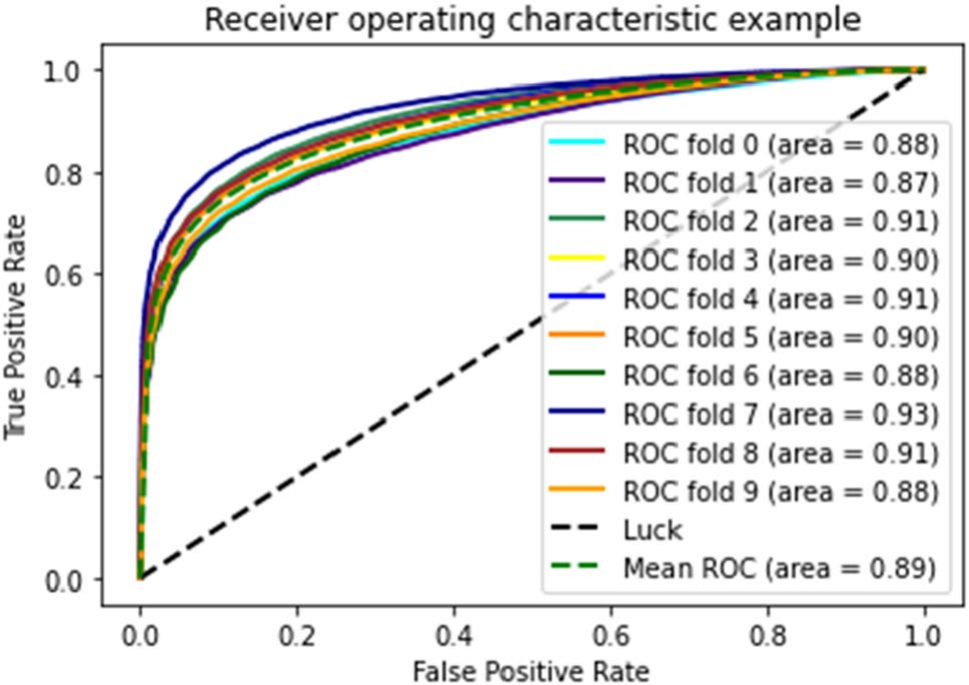
ROC curve of 10-FCV applied on GNB.

**Figure 20:**
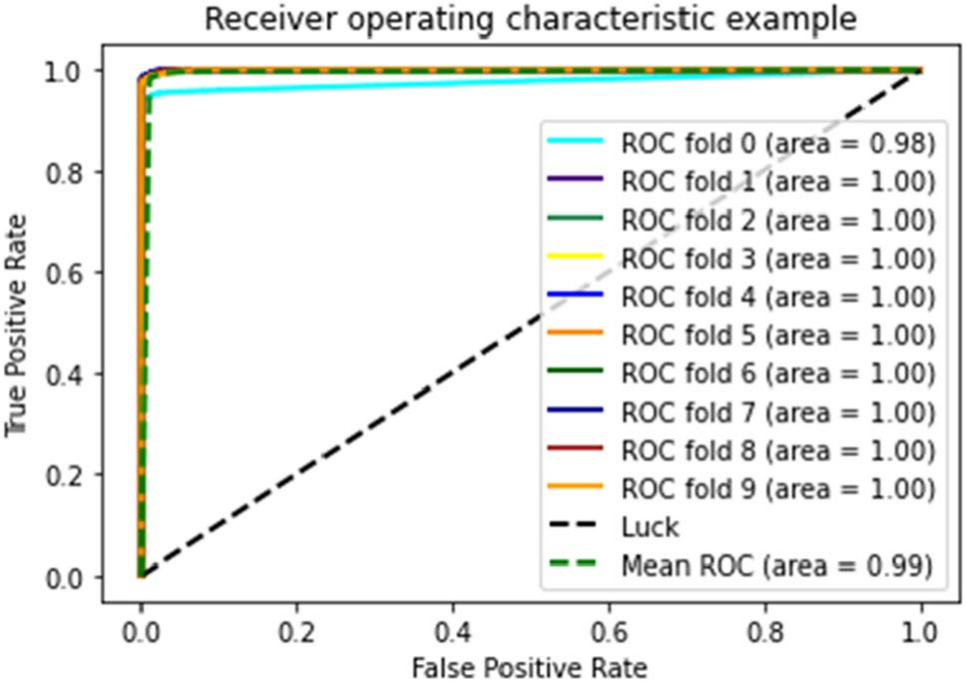
ROC curve of 10-FCV applied on RF.

Figures 18, 19, and 20 show that medical study gathered after applying tenfold cross validations on DT, GNB, and RF, respectively, for the proposed model.

**Figure 21:**
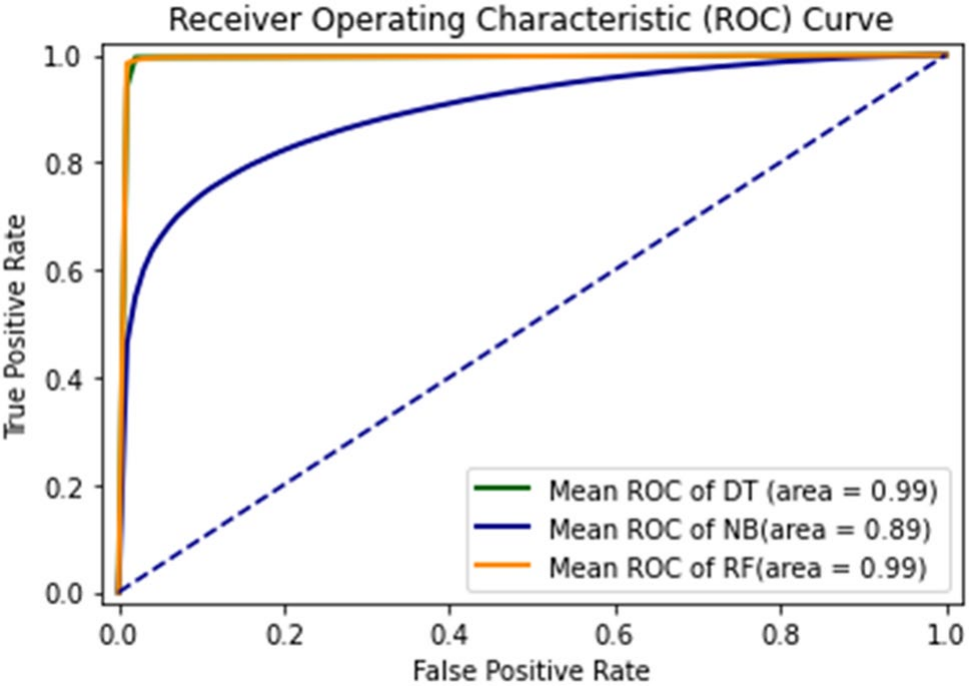
Combine the ROC curve of 10-FCV.

As shown in the figures, there are ten results for each testing technique. Tenfold cross-validation classifies the training set into ten groups^52^. And for each group, different results are gathered, and then the average is calculated. This technique is used to avoid model overfitting. This validation technique equally distributes the training set, and for every iteration, the results are different from the previous one, as shown in the ROC curves. The Combined ROC Curve of 10-FCV is shown in Fig. 21.

In Fig. 21, the combined ROC curve of maximum values is taken from each DT, GNB, and RF.

## Discussion

Breast adenocarcinoma is the most common type of cancer in women. Different types have been proposed to detect and treat this breast cancer in the past. Some researchers also present computational studies to predict breast adenocarcinoma, as discussed in the literature review section. But in those computational studies, the researcher uses small with a smaller number of entries for their research. Most of them used only one machine learning technique. The proposed technique shows the best possible results for the early detection of carcinogenic mutations in Breast Adenocarcinoma using three machine learning algorithms. An extensive dataset that includes 99 gene sequences with 6170 mutations, from 4127 human samples makes an excellent consideration dataset for this study covering all the possible scenarios. The best test data techniques for such datasets are implemented for this study. Each evaluation method has its accuracy, specificity, sensitivity, and Mathew's correlation coefficient. The ROC curves of each evaluation method are discussed in the results section. According to the AUC classification, all the accuracy results fall in the excellent category. Most results of ROC curves are on the upper side of the diagonal (50%). The decision tree shows the accuracy of 99% for each evaluation method. Gaussian Naïve Bayes gives the accuracy of 81% for the self-consistency test and independent set test and 85% for the tenfold cross-validation test. Random Forest shows 97%, 95%, and 92% accuracy for the self-consistency test, independent set test, and tenfold cross-validation test, respectively.

## Conclusion

Breast adenocarcinoma is the most common cancer in women. This study puts a positive effort into the identification of carcinogenic mutations which cause breast adenocarcinoma. Three machine learning algorithms, Decision Tree, Gaussian Naïve Bayes, and Random Forest, are applied to three types of evaluation methods independent set testing, Self-consistency testing, and tenfold cross-validation test. Accuracy, specificity, sensitivity, and Mathew’s correlation coefficient are calculated for these evaluation methods. Decision tree algorithms obtain the best accuracy of 99% for each evaluation method. All the evaluation methods’ accuracy gives results above the diagonal (50%) of the AUC value.

This study put a positive effort into identifying breast adenocarcinoma using different machine learning algorithms with a huge dataset. In the future, there may be a system that uses a more extensive dataset use for the current study and gives better results than the proposed system using different computational techniques.

## Supplementary Information

Supplementary Information.

## Data Availability

All data generated or analyzed during this study are included in this published article [and its supplementary information files].

## References

[1] Smith TJ (2013). Breast cancer surveillance guidelines. J. Oncol. Pract..

[2] Biopsy. *Cancer.Net* (2020). https://www.cancer.net/navigating-cancer-care/diagnosing-cancer/tests-and-procedures/biopsy (Accessed 23 April 2022).

[3] Fitzgerald DM, Rosenberg SM (2019). What is mutation? A chapter in the series: How microbes “jeopardize” the modern synthesis. PLoS Genet..

[4] Tolosa S, Sansón JA, Hidalgo A (2019). Theoretical study of adenine to guanine transition assisted by water and formic acid using steered molecular dynamic simulations. Front. Chem..

[5] Jackson SP, Bartek J (2009). The DNA-damage response in human biology and disease. Nature.

[6] Pegg AE (2011). Multifaceted roles of alkyltransferase and related proteins in DNA repair, DNA damage, resistance to chemotherapy, and research tools. Chem. Res. Toxicol..

[7] Zhu X, Lee H, Perry G, Smith MA (2007). Alzheimer disease, the two-hit hypothesis: An update. Biochim. et Biophys. Acta Mol. Basis Dis..

[8] Zhu X, Raina AK, Perry G, Smith MA (2004). Alzheimer's disease: The two-hit hypothesis. Lancet Neurol..

[9] Mohammed SA, Darrab S, Noaman SA, Saake G (2020). Analysis of breast cancer detection using different machine learning techniques. Data Mining Big Data..

[10] Garber J (2003). Implications of genetic information at breast cancer diagnosis. The Breast.

[11] Winchester DJ, Winchester DJ (2006). Breast Cancer.

[12] *Breast Cancer Treatment (Adult) (PDQ—ncbi.nlm.nih.gov)*. https://www.ncbi.nlm.nih.gov/books/NBK65969/. (Accessed 27 April 2022).

[13] Holm NV, Hauge M, Harvald B (1980). Etiologic factors of breast cancer elucidated by a study of unselected twins2. J. Natl. Cancer Inst..

[14] Williams WR, Anderson DE, Rao DC (1984). Genetic epidemiology of breast cancer: Segregation analysis of 200 Danish pedigrees. Genet. Epidemiol..

[15] Newman B, Austin MA, Lee M, King MC (1988). Inheritance of human breast cancer: Evidence for autosomal dominant transmission in high-risk families. Proc. Natl. Acad. Sci..

[16] Houlston RS, McCarter E, Parbhoo S, Scurr JH, Slack J (1992). Family history and risk of breast cancer. J. Med. Genet..

[17] Cancer driver mutations in breast adenocarcinoma. *IntOGen.* https://intogen.org/search?cancer=BRCA. (Accessed 24 April 2022).

[18] Pon JR, Marra MA (2015). Driver and passenger mutations in cancer. Annu. Rev. Pathol..

[19] Perou CM (2000). Molecular portraits of human breast tumours. Nature.

[20] Vaka AR, Soni B, Sudheer Reddy K (2020). Breast cancer detection by leveraging machine learning. ICT Express.

[21] Yue W, Wang Z, Chen H, Payne A, Liu X (2018). Machine learning with applications in breast cancer diagnosis and prognosis. Designs.

[22] Bazazeh, D. & Shubair, R. Comparative study of machine learning algorithms for breast cancer detection and diagnosis. In *2016 5th International Conference on Electronic Devices, Systems and Applications (ICEDSA)*. 10.1109/icedsa.2016.7818560 (2016).

[23] Khourdifi, Y. & Bahaj, M. Feature selection with fast correlation-based filter for breast cancer prediction and classification using machine learning algorithms. In *2018 International Symposium on Advanced Electrical and Communication Technologies (ISAECT)*. 10.1109/isaect.2018.8618688 (2018).

[24] Kharya S, Soni S (2016). Weighted naive Bayes classifier: A predictive model for breast cancer detection. Int. J. Comput. Appl..

[25] Malebary SJ, Khan YD (2021). Evaluating machine learning methodologies for identification of cancer driver genes. Sci. Rep..

[26] *Ensembl Genome Browser 106.* https://asia.ensembl.org/ (Accessed 24 April 2022).

[27] Generating word cloud in python. *GeeksforGeeks* (2021). https://www.geeksforgeeks.org/generating-word-cloud-python/#:~:text=Word%20Cloud%20is%20a%20data,highlighted%20using%20a%20word%20cloud. (Accessed 24 April 2022).

[28] Kaur P, Gosain A (2017). Comparing the behavior of oversampling and undersampling approach of class imbalance learning by combining class imbalance problem with noise. Adv. Intell. Syst. Comput..

[29] Chawla NV, Bowyer KW, Hall LO, Kegelmeyer WP (2002). Smote: Synthetic minority over-sampling technique. J. Artif. Intell. Res..

[30] Shah AA, Khan YD (2020). Identification of 4-carboxyglutamate residue sites based on position Based Statistical Feature and multiple classification. Sci. Rep..

[31] Zhu H, Shu H, Zhou J, Luo L, Coatrieux JL (2007). Image analysis by discrete orthogonal dual Hahn Moments. Pattern Recogn. Lett..

[32] Sohail MU, Shabbir J, Sohil F (2019). Imputation of missing values by using raw moments. Stat. Trans. New Ser..

[33] Butt AH, Khan YD (2020). Canlect-pred: A cancer therapeutics tool for prediction of Target Cancerlectins using experiential annotated proteomic sequences. IEEE Access.

[34] Barukab O, Khan YD, Khan SA, Chou K-C (2019). iSulfoTyr-PseAAC: Identify tyrosine sulfation sites by incorporating statistical moments via Chou’s 5-steps rule and pseudo components. Curr. Genomics.

[35] Navada, A., Ansari, A. N., Patil, S. & Sonkamble, B. A. Overview of use of decision tree algorithms in machine learning. In *2011 IEEE Control and System Graduate Research Colloquium*. 10.1109/icsgrc.2011.5991826 (2011).

[36] Abid Mahmood Malik Hafiz (2022). Complex Network Formation and Analysis of Online Social Media Systems. Computer Modeling in Engineering & Sciences.

[37] Abid Mahmood Malik Hafiz (2022). Analysis of social media complex system using community detection algorithms. Int. J. Comput. Digit. Syst.

[38] *Which Test is More Informative?—homes.cs.washington.edu*. https://homes.cs.washington.edu/~shapiro/EE596/notes/InfoGain.pdf (Accessed 23 April 2022).

[39] Decision tree algorithm, explained. *KDnugget.* https://www.kdnuggets.com/2020/01/decision-tree-algorithm-explained.html (Accessed 24 April 2022).

[40] Salmi N, Rustam Z (2019). Naïve bayes classifier models for predicting the colon cancer. IOP Conf. Ser. Mater. Sci. Eng..

[41] Kaviani P, Dhotre MS (2017). Short survey on naive Bayes algorithm. Int. J. Adv. Eng. Res. Dev..

[42] Gu J (2018). Recent advances in convolutional neural networks. Pattern Recogn..

[43] Maheswari S, Pitchai R (2019). Heart disease prediction system using decision tree and naive Bayes algorithm. Curr. Med. Imaging Form. Curr. Med. Imaging Rev..

[44] Awais M, Hussain W, Rasool N, Khan YD (2021). iTSP-PseAAC: Identifying tumor suppressor proteins by using fully connected neural network and PseAAC. Curr. Bioinform..

[45] Schott, M. Random Forest algorithm for machine learning. *Medium* (2020). https://medium.com/capital-one-tech/random-forest-algorithm-for-machine-learning-c4b2c8cc9feb (Accessed 24 April 2022).

[46] Schonlau M, Zou RY (2020). The Random Forest algorithm for statistical learning. Stata J. Promot. Commun. Stat. Stata.

[47] Trevethan R (2017). Sensitivity, specificity, and predictive values: Foundations, pliabilities, and pitfalls in research and Practice. Front. Public Health.

[48] van Stralen KJ (2009). Diagnostic methods I: Sensitivity, specificity, and other measures of accuracy. Kidney Int..

[49] Lalkhen AG, McCluskey A (2008). Clinical tests: Sensitivity and specificity. Contin. Educ. Anaesth. Crit. Care Pain.

[50] Kulkarni A, Chong D, Batarseh FA (2020). Foundations of data imbalance and solutions for a data democracy. Data Democracy..

[51] Hoo ZH, Candlish J, Teare D (2017). What is an ROC curve?. Emerg. Med. J..

[52] Sengar, P. P., Gaikwad, M. J. & Nagdive, A. S. Comparative study of machine learning algorithms for breast cancer prediction. In *2020 Third International Conference on Smart Systems and Inventive Technology (ICSSIT)*. 10.1109/icssit48917.2020.9214267 (2020).

